# IL-18 production upon s100 stimulation is reduced in active sJIA patients compared to sJIA patients in remission and healthy controls

**DOI:** 10.1186/1546-0096-12-S1-O24

**Published:** 2014-09-17

**Authors:** Nienke Ter Haar, Dirk Holzinger, Wilco de Jager, Rianne Scholman, Sytze de Roock, Bas Vastert

**Affiliations:** 1 Laboratory for Translational Immunology, University Medical Center Utrecht, Utrecht, Netherlands; 2 Institute of Immunology, University Hospital Muenster, Muenster, Germany; 3 Pediatric Immunology and Rheumatology, University Medical Center Utrecht, Utrecht, Netherlands

## Introduction

Systemic onset Juvenile Idiopathic Artritis (sJIA) is considered to be an autoinflammatory disease. The S100-proteins S100A8, S100A9 and S100A12 in serum and interleukin (IL)-18 in plasma are extremely elevated in sJIA patients and have been proposed to be useful biomarkers for diagnosis, disease activity and response to therapy. Moreover, sJIA patients seem to have a defective IL-18 NK cell axis as NK cell lytic function is impaired in spite of high IL-18 levels. However, it is still unknown how the inflammatory S100 proteins and IL-18 relate to each other, and to the defective NK cell lytic function.

## Objectives

Our aim is to study the relation between S100 proteins and IL-18 in sJIA.

## Methods

sJIA patients are prospectively followed and sampled during active disease, during treatment and in remission. Peripheral blood mononuclear cells (PBMCs) from sJIA patients during active disease and during remission and PBMCs from healthy controls (HCs) were stimulated with S100A8, S100A9, S100A12 and LPS for four hours. As a 2^nd^ signal for NLRP3 inflammasome activation, ATP was added during the last 30 minutes. To investigate signalling pathways of the S100 proteins, TLR4 and RAGE were blocked by adding CLI-095 and anti-RAGE antibody, respectively. Cytokine levels in supernatant were measured by Luminex; cell frequencies and TLR-expression were analyzed by flow cytometry. Caspase 1-activity was measured with a colorimetric assay (RnD). Further, mRNA levels of NLRP3, IL-1b and IL-18 after S100 stimulation were assessed by qPCR.

## Results

Stimulation of PBMC with S100A8, S100A9 and S100A12 resulted in cytokine production of IL-1b, IL-18 as well as IL-6 and TNF-a. The addition of ATP during the last 30 minutes of stimulation further enhanced IL-1b and IL-18 levels. Blocking TLR4 by adding CLI-095 decreased cytokine production to near normal levels, while blocking RAGE did not have an effect on cytokine production.

When compared to healthy controls, PBMCs from sJIA patients produced less IL-18 upon S100 stimulation. The addition of ATP enhanced this differential effect. Four paired samples of active disease and remission were analyzed; PBMCs from active patients were less responsive to S100 stimulation compared to PBMCs from the same patient while in remission. There were no significant differences in cell subset frequencies, viability of the cells or TLR4 expression in these patients.

## Conclusion

S100A8, S100A9 and S100A12 serve as a first signal to establish NLRP3 inflammasome activation in sJIA patients and healthy controls. This effect is TLR4-dependent. Interestingly, S100 proteins induce less IL-1b and IL-18 production in PBMC from active sJIA patients compared to sJIA patients in remission and healthy controls. If and how these findings relate to involved celltypes like neutrophils, monocytes, T cells and NK cells is currently under investigation.

## Disclosure of interest

None declared.

